# Peptides Derived From Reactive Center Loops Inhibit Digestive Trypsin‐Like Enzymes in Lepidopteran Pests

**DOI:** 10.1002/arch.70123

**Published:** 2026-01-09

**Authors:** Daniel Guimarães Silva Paulo, Julia Renata Schneider, Yaremis Meriño‐Cabrera, Wesley Borges Wurlitzer, Rafael Júnior de Andrade, Ian Lucas Batista Santos, Maria Clara Neves Gomes Rodrigues, Rafael de Almeida Barros, Neilier Rodrigues da Silva Júnior, Milena Godoi Lima, Cibele Cunha Vilela, Noeli Juarez Ferla, Humberto Josué Ramos de Oliveira, Maria Goreti de Almeida Oliveira

**Affiliations:** ^1^ Department of Entomology Federal University of Viçosa Viçosa Minas Gerais Brazil; ^2^ Institute of Biotechnology Applied to Agriculture (BIOAGRO‐UFV) Viçosa Minas Gerais Brazil; ^3^ Acarology Laboratory University of Vale do Taquari Lajeado Brazil; ^4^ Research Group on Agroindustrial Processes and Sustainable Development (PADES) University of Sucre Sincelejo Colombia; ^5^ Department of Biochemistry and Molecular Biology Federal University of Viçosa Viçosa Minas Gerais Brazil

**Keywords:** *Anticarsia gemmatalis*, glycine max, inhibitory constant, molecular docking, plants, reactive center loops

## Abstract

Soybean yield is often reduced by pest attacks. Among these, *Anticarsia gemmatalis* Hübner (Lepidoptera: Noctuidae) stands out as one of the most important defoliating pests of soybean. Therefore, the development of new bioinsecticides targeting Lepidopteran pests is an urgent need. Protease inhibitors (PIs) have emerged as promising molecules in this context. In this study, we designed four peptides (TGPCK, TGPCR, AVIMK, and AVIMR) inspired by the reactive center loops of BPTI and SKTI to assess their potential as competitive inhibitors of trypsin‐like proteases in *A. gemmatalis*. In silico and kinetic analyses revealed that peptide binding affinity was influenced by specific chemical interactions, with pi‐sigma bonds correlating with higher affinity for AVIMK, while alkyl/pi‐alkyl and C‐H bonds were associated with lower affinity for AVIMR and TGPCK. Key residues (His57, Asp102, Ser195, Asp189, S195, and G197) played a crucial role in ligand binding. Enzyme inhibition assays confirmed that all peptides acted as competitive inhibitors of *A. gemmatalis* trypsin‐*glen* proteases, with TGPCK displaying the highest efficacy. These findings highlight BPTI‐derived peptides as potential candidates for future pest management strategies. Further studies should evaluate their effects when applied to plants, considering possible metabolic interactions and phytotoxicity.

## Summary

- Molecular docking showed that peptides based on BPTI presented higher affinities than SKTI based.
- Binding with conserved residues at the active site of lepidopteran trypsins is correlated with higher affinity.
- The pentapeptide TGPCK presented a great inhibitory capacity of *A. gemmatalis* trypsins.

## Graphical abstract

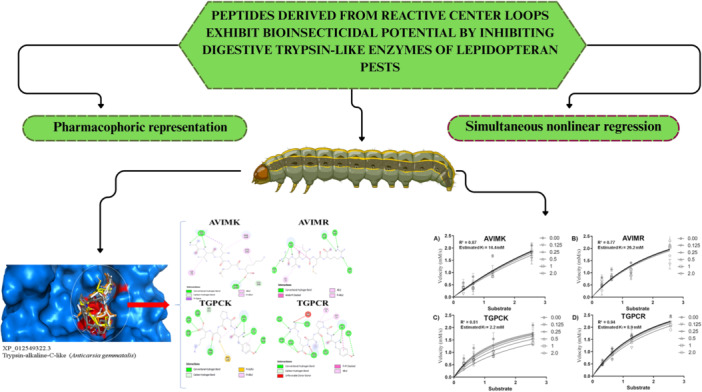

## 1 Introduction

Soybeans (Glycine max (L.) Merr.) are one of the most important agricultural crops worldwide, particularly due to their economic and social relevance in the production of vegetable oil and protein‐rich feed. However, soybean yield is often compromised by biotic factors, especially the action of insect pests. Among them, *Anticarsia gemmatalis* Hübner (Lepidoptera: Noctuidae) stands out as one of the main defoliating pests of soybean (Silvestre et al. 2022), causing significant yield losses when not properly controlled (Sultana et al. 2021).

Chemical control remains the most widely used strategy for managing this pest. Nevertheless, the recurrent use of synthetic insecticides has led to increasing challenges, such as the selection of resistant insect populations (Nauen and Steinbach 2016), adverse environmental impacts, and risks to human health (Braga et al. 2020; Pimentel and Burgess 2014). This scenario has driven the search for more sustainable alternatives that can be integrated into Integrated Pest Management (IPM) programs (Jacquet et al. 2022; Vasantha‐Srinivasan et al. 2025).

Insect digestive enzymes are promising targets for developing novel biopesticides. Lepidopteran pests rely primarily on serine proteases, particularly trypsin‐ and chymotrypsin‐like enzymes, for protein digestion (da Silva Júnior et al. (2020); Napoleão et al. 2019a). Exposure to trypsin‐*like* protease inhibitors often disrupts insect development by reducing amino acid availability and causing metabolic deregulation, ultimately impairing survival and reproduction (Pandey et al. 2022). However, despite their insecticidal potential, protease inhibitors have yet to be widely adopted for pest control due to adaptive countermeasures in insects, such as the overexpression of insensitive proteases and endogenous proteolytic cleavage of protease inhibitors (Singh et al. 2020). Additional challenges include the need for prolonged exposure to achieve insecticidal effects and limited environmental stability (Singh et al. 2020; Zhu‐Salzman and Zeng 2015).

Plants naturally produce protease inhibitors as a defense mechanism against herbivores (Divekar et al. 2022). These inhibitors are induced upon insect attack through elicitors in insect saliva and mechanical damage, which trigger phytohormonal signaling involving jasmonic acid (JA), salicylic acid (SA), and abscisic acid (ABA) (Sultana et al. 2021). Chronic exposure to plant‐derived PIs negatively affects herbivore performance (Hartl et al. 2011). However, most natural protease inhibitors are large protein molecules that are susceptible to degradation by non‐target proteases in the insect midgut, limiting their effectiveness. Their inhibitory activity is mediated by small regions known as reactive center loops (RCLs) (Zhou et al. 2001a). To overcome these limitations, RCL‐derived peptides with improved stability and biological activity can be developed. Previous studies have demonstrated that RCL‐derived peptides from serine protease inhibitors (SERPINS) exhibit enhanced biological functions compared to their full‐length proteins (Ambadapadi et al. 2016). Similarly, small scaffold peptides with inhibitory activity have been shown to impair insect fitness (Fiandra et al. 2009; Spit et al. 2012; Saikhedkar et al. 2018a; Saikhedkar et al. 2019; Patarroyo‐Vargas et al. 2020; Meriño‐Cabrera et al. 2020).

Developing non‐host‐derived RCL peptides that can inhibit vital insect digestive proteases offers a promising strategy for overcoming insect adaptations that have evolved through coevolution with plants. Optimized peptide‐based formulations could enhance the efficacy and stability of protease inhibitors in pest management (Nowicki et al. 2021). Additionally, genetically engineered crops expressing multiple inhibitory RCL peptides could provide a durable and sustainable approach to insect control.

In this study, we designed and evaluated five‐residue peptides derived from the RCLs of Bovine Pancreatic Trypsin Inhibitor (BPTI) and Soybean Kunitz Trypsin Inhibitor (SKTI) for their ability to competitively inhibit trypsin‐*like* proteases from Lepidoptera pests. By identifying peptides that effectively target vital digestive enzymes, this study contributes to the development of sustainable protein‐based biopesticides for agricultural pest control.

In this investigation, we assessed the potential of pentapeptides derived from protease inhibitors as bioactive agents for the management of *A. gemmatalis*, one of the principal pests affecting soybean crops, with the objective of advancing more sustainable and effective strategies for integrated pest management.

## 2 Material and Methods

### 2.1 Study Subject

The species *Anticarsia gemmatalis*, an important defoliating pest of soybean in South America, was selected due to its agronomic impact.

### 2.2 Peptide Design

Four pentapeptides were designed based on the reactive center loops (RCLs) of Bovine Pancreatic Trypsin Inhibitor (BPTI) and Soybean Kunitz Trypsin Inhibitor (SKTI). Two peptides (AVIMK and AVIMR) were derived from SKTI, based on docking analyses of SKTI interacting with a trypsin‐like alkaline isoform identified in the proteomic profile of *Anticarsia gemmatalis* Hübner (Lepidoptera: Noctuidae) (da Silva Júnior et al. (2020)). Two additional peptides (TGPCK and TGPCR) were designed from the BPTI sequence (NCBI accession: 1CBW), focusing on its known trypsin‐interacting region. In all cases, the fifth residue was replaced with either arginine (Arg) or lysine (Lys) to optimize binding to the S1 pocket of trypsin‐*like* enzymes. Three‐dimensional structures of the peptides were modified using Biovia Discovery Studio® software and saved in PDBQT format for docking analysis.

### 2.3 Trypsin‐*Like* Enzymes From Lepidopteran Pests

**Figure 1:**
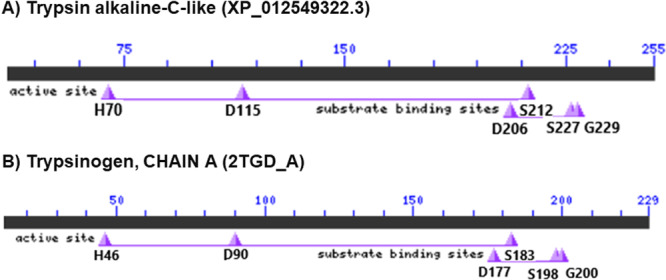
(A) Trypsin alkaline‐C‐like enzyme (XP_012549322.3). (B) Trypsinogen, chain A (PDB: 2TGD_A). Schematic representation based on BLASTP alignment between a lepidopteran alkaline trypsin‐like enzyme (XP_012549322.3) and bovine trypsinogen. Conserved catalytically relevant residues (His, Asp, and Ser) are highlighted according to chymotrypsinogen numbering, supporting the equivalence of the active site architecture (Hedstrom 2002d).

FASTA sequences of eight trypsin‐*like* enzymes from *A. gemmatalis, Helicoverpa armigera* (Hübner), *Spodoptera litura* (Fabricius), and *Spodoptera frugiperda* (Smith) (Lepidoptera: Noctuidae) were retrieved from the NCBI Protein Database (https://www.ncbi.nlm.nih.gov/protein/; accessed December, 2025) (Table 1). The 3D structures of these enzymes were predicted using the Phyre2 platform (Kelley et al. 2015) and validated through structure quality assessments, including ProSa‐web (Protein Structure Analysis) (https://prosa.services.came.sbg.ac.at/prosa.php), ERRAT (http://services.mbi.ucla.edu/ERRAT/), and Ramachandran plot analysis (RAMPAGE) (http://mordred.bioc.cam.ac.uk/~rapper/rampage.php). Catalytic residues were annotated using chymotrypsinogen numbering, as determined by NCBI BLASTP (accessed in December, 2025), and aligned with bovine trypsinogen. Figure 1 presents a representative alignment between bovine trypsinogen and a trypsin alkaline C‐like isoform from *A. gemmatalis*.

**Table 1:** Trypsin‐like sequences obtained from the NCBI database.

Denomination	Species	NCBI Identifier
AgTry1	*A. gemmatalis*	AWL83213.1
AgTry2		XP_012549322.3
SfTry1	*S. frugiperda*	XP_035433891.1
SfTry2		XP_035433885.1
HaTry1	*H. armigera*	XP_021200143.1
HaTry2		XP_021199089.1
SlTry1	*S. litura*	XP_022815737.1
SlTry2		XP_022814598.1

### 2.4 Molecular Docking and Interaction Analysis

Energy‐minimized peptids and enzyme structures (PDBQT files) were loaded into PyRx (version 0.8) using the AutoDock (version 1.1.2) Vina Wizard for molecular docking. Default grid parameters were maintained. For each enzyme, the top‐ranked docking pose per peptide was selected based on binding affinity. A total of eight docking poses were analyzed per peptide across the trypsin‐like enzymes. The best binding poses and associated affinity energies were selected and further examined for interaction types and ligand orientation. Pharmacophore analyses were conducted using DS Visualizer 4.0 (Discovery Studio, version 4.0).

Binding affinity values (kcal mol^−1^) were subjected to one‐way ANOVA followed by Duncan's post‐hoc test. Additionally, Pearson correlation coefficients were used to explore relationships between binding affinity and (i) the number of interactions, (ii) interaction types, and (iii) binding to conserved active‐site residues.

### 2.5 Inhibitory Constant Assessing (*K*_i_)

Apparent *K_i_* values were determined by measuring trypsin‐*like* activity in crude midgut extracts from *A. gemmatalis* larvae. Fifth‐instar larvae, reared according to (Greene et al. 1976) were dissected to isolate the gut, which was lysed in 1 ml of acidified water (pH 3.0, 1 M HCl) using a TissueLyser II (Qiagen®). The lysate was centrifuged at 10,000 *g* (4° C, 30 min), and the supernatant was used for protein quantification via the Bradford assay.

Trypsin activity was assayed using l‐BApNA as the substrate at four concentrations (2.56, 1.28, 0.64, and 0.32 mM) in the presence of five peptide concentrations (0, 0.25, 0.5, 1.0, and 2.0 mM). Reactions (250 μL total volume) were performed in 96‐well plates containing 0.05 M Tris‐HCl (pH 8.0), 20 mM CaCl_2_, 30 μg of midgut extract, peptide, and substrate. Absorbance at 410 nm was recorded for 2 min using a SpectraMax M2 spectrophotometer. *K_i_* values and inhibition models (competitive, non‐competitive, mixed) were calculated using the Nonlinear Regression (Curve Fitting) module in GraphPad Prism 5.0.

## 3 Results

### 3.1 Binding Free Energy Analysis

**Figure 2:**
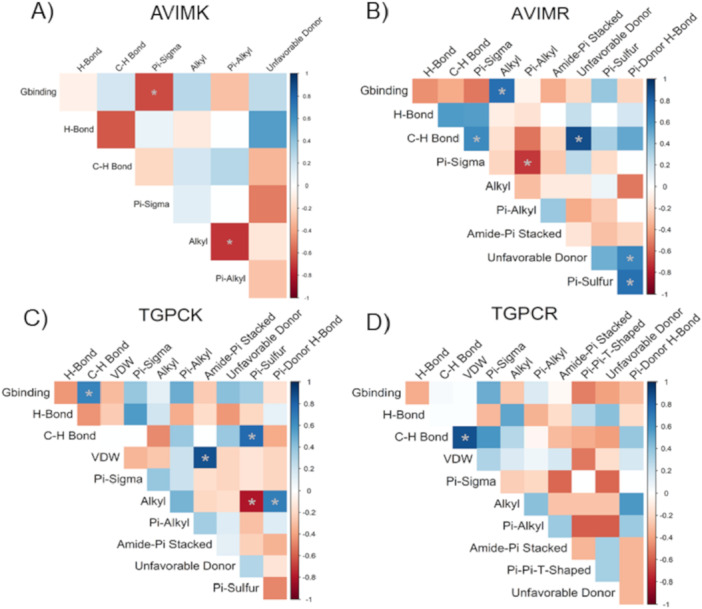
Heatmap for pearson's correlation (*r*) analysis between ΔG_BINDING and chemical bond types involved in complex formation between the peptides (A) AVIMK, (B) AVIMR, (C) TGPCK, and (D) TGPCR and lepidopteran alkaline trypsin‐like enzymes. Asterisks indicate statistically significant correlations (*p < 0.05).

The impact of specific chemical interactions on the binding free energy Δ*G*_BINDING_ between peptides and trypsin‐like enzymes was assessed using Pearson correlation (*r*) and visualized in heatmaps (Figure 2). Among SKTI‐derived peptides, a higher number of pi‐sigma interactions between AVIMK and lepidopteran trypsins correlated with more favorable Δ*G*_BINDING_ values. In contrast, alkyl interactions in AVIMR‐trypsin complexes were associated with weaker binding (i.e., higher Δ*G*_BINDING_) (Figure 2A, B). For BPTI‐derived peptides, a strong positive correlation was observed between C‐H bonds and higher Δ*G*_BINDING_ in TGPCK complexes, indicating weaker interactions. In contrast, TGPCR showed no significant correlation between any specific interaction type and binding energy (Figure 2C, D).

**Figure 3:**
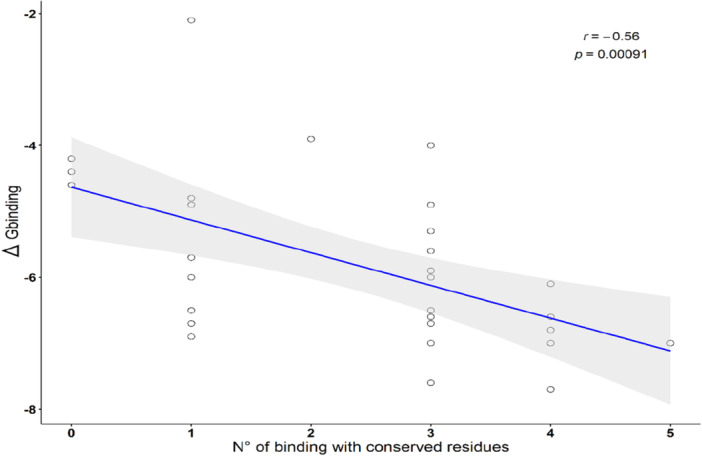
Correlation analysis representation between n° of binding with conserved residues and the binding free energy. The Pearson correlation coefficient (*r*) and corresponding p‐value are shown in the plot. Opened circles represent the binding free energy and the number of interactions with active site residues, i.e., His57, Asp102, Ser195, Asp189, S195, and G197 (Chymotrypsinogen numbering) for each of the complexes formed (32) between lepidopteran trypsins and the peptides. The solid blue line represents linear regression, and the gray shaded area indicates the 95% confidence interval (CI).

On average, BPTI‐derived peptides and the reference substrate l‐BApNA displayed lower (i.e., more favorable) binding energies than SKTI‐derived peptides across the panel of Lepidoptera trypsins (Table 2). The total number of peptide‐enzyme interactions did not significantly correlate with binding affinity (*r* = −0.289; t' test‐5% = −1.655; *p* > 0.05). However, binding to conserved catalytic residues was strongly correlated with binding strength. Specifically, the number of interactions with His57, Asp102, Ser195, Asp189, S195, and Gly197 (chymotrypsinogen numbering) showed a strong negative correlation with Δ*G*_BINDING_ (*r* = −0.558; t' test‐5% = −3.679; *p* < 0.00045), suggesting that these contacts are key contributors to peptide affinity (Figure 3).

**Table 2:** Binding free energy (kcal/mol) of the complex formation between Peptides/BApNA and Alkaline Trypsin‐like enzymes of lepidopteran insects.

	Ligands				
Receptor	AVIMK	AVIMR	TGPCK	TGPCR	BApNA
AgTry1	−4.2	−4.4	−4.9	−4.6	−6.5
AgTry2	−5.9	−6.0	−6.5	−6.9	−5.8
SfTry1	−6.5	−6.7	−7.6	−7.7	−7.7
SfTry2	−4.9	−6.1	−6.8	−7.0	−6.9
HaTry1	−6.5	−6.7	−7.0	−6.6	−7.9
HaTry2	−4.8	−2.1	−5.7	−6.0	−7.3
SlTry1	−5.3	−5.6	−6.6	−7.0	−6.0
SlTry2	−3.9	−4.9	−4.0	−6.6	−5.4
Duncan's test	−5.25 ± 0.35 A	−5.31 ± 0.54 A	−6.14 ± 0.42B	−6.55 ± 0.33B	−6.69 ± 0.32B

### 3.2 Pharmacophore Profiles

**Figure 4:**
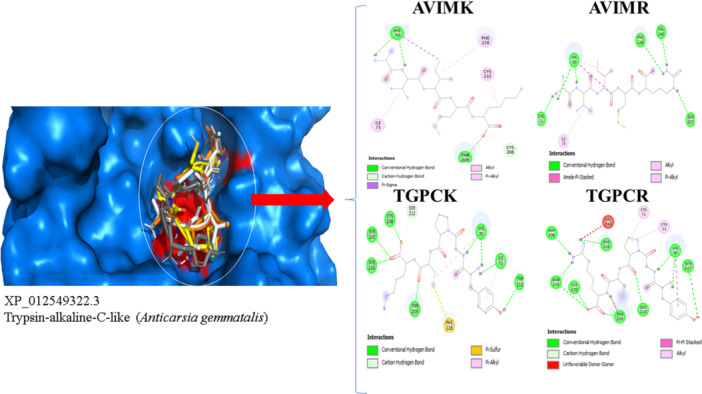
Pharmacophoric interaction profiles of the peptides AVIMK, AVIMR, TGPCK, and TGPCR with lepidopteran alkaline trypsin‐like enzymes. Pie charts represent the relative contribution (%) of each interaction type involved in complex formation, including hydrogen bonds, C–H bonds, alkyl/π.

**Figure 5:**
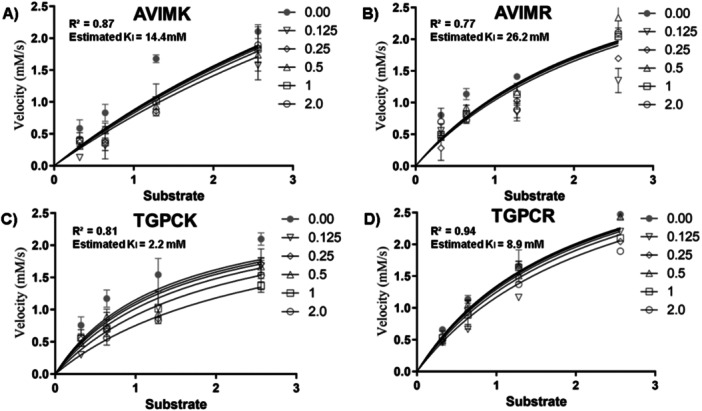
Kinetic analysis of trypsin inhibition by the peptides (A) AVIMK, (B) AVIMR, (C) TGPCK, and (D) TGPCR. Reaction velocity (mM/s) is shown as a function of substrate concentration (mM).

All peptides formed at least one interaction with conserved residues in the catalytic triad, except for AVIMK, AVIMR, and TGPCR when docked to the 1TryAg isoform (Figure 4; Supporting Material 1). Despite this, notable differences in interaction types were observed among peptides (Figure 5). Overall, H‐bonds, C‐H bonds, and alkyl/Pi alkyl interactions predominated. Notably, TGPCR exhibited a higher number of C‐H bonds compared to other peptides. Peptides containing lysine (K) at the P1 position formed significantly more pi‐sigma bonds, which were correlated with increased binding affinity (Figure S2).

Unfavorable interactions were more frequent in TGPCR complexes, with three predicted unfavorable contacts across the trypsin models. In contrast, TGPCK and AVIMK each exhibited only one unfavorable binding event, whereas AVIMR had two. These data suggest that both the nature and positioning of terminal residues significantly influence peptide–enzyme compatibility.

### 3.3 Inhibition Kinetics

The apparent inhibition constants (*K_i_*) determined from kinetic assays using crude midgut extracts of *A. gemmatalis* indicated that the BPTI‐derived peptides were more effective inhibitors than SKTI‐derived ones. TGPCK showed the highest potency (*K_i_* = 2.2 mM), followed by TGPCR (8.8 mM), AVIMK (14.4 mM), and AVIMR (26.2 mM). These data indicate that the TGPCK peptide may reduce protein digestibility in the midgut of *A. gemmatalis*, negatively affecting its larval development, which is desirable in the context of soybean pest control.

For all peptides, the best fit to the inhibition model was obtained under competitive inhibition, as evidenced by *R* ^2^ values of 0.94 (TGPCR), 0.87 (AVIMK), 0.81 (TGPCK), and 0.77 (AVIMR) (Figure 5). These results support the potential of BPTI‐based pentapeptides, especially TGPCK, as potent competitive inhibitors of trypsin‐like enzymes in *A. gemmatalis*.

## 4 Discussion

Protease inhibitors (PIs) play a pivotal role in plant defense against herbivores by inhibiting digestive enzymes in the insect gut, thereby impairing nutrient assimilation and reducing pest fitness (Divekar et al. 2022). These inhibitors, along with other protein‐based defenses such as lectins, have been extensively studied for their insecticidal potential and mode of action in a variety of plant–insect systems (Murdock and Shade 2002). However, the biosynthesis of these compounds imposes significant metabolic costs (Neilson et al. 2013; Cunha et al. 2023). While the capacity to produce PIs confers an adaptive advantage, understanding the trade‐offs involved, especially in agricultural contexts, is crucial. Upon herbivore attack, resource allocation shifts from primary metabolism to defense‐related pathways, reducing investment in growth, reproduction, and yield, as resources are redirected toward the synthesis of defense metabolites such as PIs.

The extensive use of synthetic insecticides to control Lepidoptera pests raises serious sustainability concerns, particularly due to their environmental impact and the widespread development of resistance in pest populations (Gould et al. 2018; Hawkins et al. 2019; Van Leeuwen et al. 2020; Saakre et al. 2024). Although biotechnological tools have enabled the development of biological formulations, most currently available biopesticides rely on a limited set of mechanisms, primarily *Bacillus thuringiensis‐derived* Cry and Vip proteins, and entomopathogenic fungi like *Metarhizium anisopliae* and *Beauveria bassiana* (Chandler et al. 2008; Umetsu and Shirai 2020). This limited diversity of modes of action underscores the urgent need for novel biological molecules with distinct insecticidal mechanisms.

In our study, the binding affinities (Δ*G*, kcal/mol) of SKTI‐derived peptides AVIMK and AVIMR were closely associated with the presence of Pi‐sigma (negatively correlated) and alkyl (positively correlated) interactions. Pi‐sigma interactions involve orbital overlap that stabilizes charged intermediates within enzyme active sites, typically enhancing ligand affinity (Arthur and Uzairu 2019). Conversely, alkyl interactions contribute to hydrophobic binding, particularly within the S2 and S3 subsites of trypsins (Hedstrom 2002a). While alkyl groups enhance flexibility and facilitate ligand accommodation, excessive flexibility or steric bulk can compromise tight binding (Hedstrom 2002b). For instance, derivatives of 5‐(alkyloxy)‐tryptamine with longer alkyl chains exhibited reduced affinity for serotonin receptors. Similarly, in the TGPCK peptide, C‐H interactions were associated with lower affinity for trypsin‐like enzymes. Although weaker than conventional hydrogen bonds, C‐H interactions can still influence ligand stability and retention (Herrebout and Suhm 2011), and their predominance may interfere with the formation of stronger hydrogen bonds.

The similar binding energies of BPTI‐derived peptides with the chromogenic substrate l‐BApNA suggest high affinity of peptides like TGPCK and TGPCR for lepidopteran trypsins, assuming this substrate accurately reflects enzymatic activity in vitro (Oliveira et al. 1993; do Amaral et al. 2022). Although SKTI‐derived peptides displayed spontaneous interaction (negative Δ*G*), their inhibitory efficiency may depend on higher peptide concentrations in physiological systems. However, it is important to note that kinetic assays were performed using crude midgut extracts, which comprise a heterogeneous mixture of trypsin‐like isoforms and other proteolytic enzymes with variable sensitivity to inhibition (de Oliveira et al. 2020; Napoleão et al. 2019b). This enzymatic complexity can attenuate apparent inhibitory effects and obscure well‐defined inhibition curves, particularly for small competitive inhibitors (Napoleão et al. 2019a; Patarroyo‐Vargas et al. 2020; da Silva Júnior et al. 2020). Moreover, competitive inhibitors with low molecular mass are readily displaced at higher substrate concentrations, particularly in assays using chromogenic substrates such as l‐BApNA, which may further reduce the visual separation of kinetic curves (Robin et al. 2018; Lopes et al. 2006). Significantly, binding affinity did not correlate with the total number of ligand‐enzyme interactions but rather with contact to specific residues within the catalytic triad (His57, Asp102, Ser195) and the S1 subsite (Asp189). These residues are critical for enzymatic function, His57, Asp102, and Ser195 form the canonical triad; Asp189 confers specificity for basic residues; and Ser195 and Gly197 stabilize the transition state during catalysis (Hedstrom 2002b; Robin et al. 2018). At first glance, this relationship may appear counterintuitive, highlighting the risk of overinterpreting interaction frequency as a proxy for functional inhibition. This is exemplified by AVIMR, which showed an elevated number of hydrogen‐bond contacts in the interaction profiles but negligible inhibitory activity in kinetic assays. This apparent discrepancy underscores a key limitation of global interaction counts as predictors of functional inhibition. Many predicted contacts may occur at peripheral or solvent‐exposed regions of the enzyme and, therefore, do not effectively block substrate access or perturb the catalytic machinery. In contrast, interactions strategically positioned within the catalytic triad and the S1 specificity pocket are more likely to promote a competitive binding mode and translate into measurable enzymatic inhibition.

Molecular docking predicted that most peptide‐trypsin complexes formed direct interactions with these key residues, suggesting a competitive inhibition mechanism. In this model, inhibitors compete with the substrate for the same binding site, and increasing substrate concentration may reduce the inhibitory effect (Robin et al. 2018). Some peptides showed predicted unfavorable interactions, possibly due to steric hindrance, which could impair binding. However, such unfavorable interactions do not necessarily indicate poor in vivo inhibition. Molecular docking tools, like PyRx do not account for receptor flexibility, induced fit effects, solvation, entropy, or complex physiological interactions, all of which can significantly influence inhibitory behavior (Taylor et al. 2002).

Our kinetic data confirm that BPTI‐derived peptides are more potent inhibitors of *A. gemmatalis* trypsin‐like enzymes than SKTI‐derived inhibitors. Among the SKTI‐derived peptides, AVIMK exhibited a weak inhibitory effect, whereas AVIMR showed a negligible inhibitory potential, with activity detectable only at the highest concentration tested. Peptides AVIMK and AVIMR exhibited high *K_i_* values, above the range typically associated with effective inhibitors, which generally span from 0.005 mM to 4.31 mM (de Almeida Barros et al. 2021; de Almeida Barros et al. 2022; Meriño‐Cabrera et al. 2022). This reduced potency may be related to their linear structure and the absence of structural constraints provided by their native proteins, which is consistent with previous studies showing that short, linear peptides lack the conformational rigidity required for optimal occupation of the catalytic site, resulting in higher apparent Ki values when compared with full‐length or structurally constrained inhibitors (Hedstrom 2002c; Saikhedkar et al. 2018b; Zhou et al. 2001b). For instance, the RCLs of SERPIN18 from *Bombyx mori* (Lepidoptera: Noctuidae) require specific conformational movements to insert into a β‐sheet of the target enzyme (Guo et al. 2015), illustrating how critical the parent protein's structure is to RCL functionality.

Among the BPTI‐derived peptides, TGPCK demonstrated the highest potency, with a K_i_ approximately four times lower than that of TGPCR, indicating stronger interaction with *A. gemmatalis* trypsins. Interestingly, while previous studies have suggested a slight preference for Arg over Lys at the P1 in shorter peptides (de Almeida Barros et al. 2021; de Almeida Barros et al. 2022), other work using octapeptides found no clear preference between these residues (Lopes et al. 2006). Our results suggest that in larger peptides, the guanidinium group of Arg may introduce steric hindrance in the active site, reducing binding efficiency. This is further supported by the greater number of predicted unfavorable contacts for TGPCR than for TGPCK.

In addition, the efficacy of these peptides must be evaluated under more realistic agronomic conditions. Application to plants may involve interactions with plant primary and secondary metabolism, potentially affecting peptide stability, bioavailability, or phytotoxicity. Assessing these factors will be essential before considering applied or field‐based strategies. Environmental factors such as proteolytic degradation, pH variation, and exposure to ultraviolet radiation may further influence peptide performance. Consequently, formulation strategies to enhance stability and persistence should be explored to improve the practical applicability of peptide‐based inhibitors.

These findings highlight the importance of both sequence composition and structural compatibility in peptide design. The detailed molecular interactions described here provide a foundation for rational engineering of more effective peptide‐based inhibitors targeting insect digestive proteases. However, because the peptides evaluated in this study showed binding potential for trypsin‐like enzymes across multiple Lepidoptera species, their specificity for pest insects warrants careful consideration. Many Lepidoptera play beneficial ecological roles, such as pollination, and non‐target inhibition of digestive proteases may represent a potential environmental risk (Krenn 2010; Goulson 2019). Therefore, future studies should include in silico and in vitro evaluations of enzymes from non‐pest species to more effectively assess selectivity and ecological safety.

## 5 Conclusions

This study demonstrates that pentapeptides derived from reactive center loops can interact with and competitively inhibit digestive trypsin‐like enzymes from *Anticarsia gemmatalis*. Among the evaluated sequences, the BPTI‐derived peptide TGPCK exhibited the highest inhibitory potential, displaying stronger binding to catalytically essential residues and a lower Ki value than the SKTI‐derived peptides.

By integrating molecular docking, pharmacophore analysis, and kinetic assays, our results indicate that inhibitory efficiency is primarily associated with interactions at the catalytic triad and the S1 specificity subsite rather than with the total number of ligand–enzyme contacts. These findings underscore TGPCK as a promising lead structure and offer a mechanistic foundation for the rational design of optimized peptide‐based inhibitors targeting insect digestive proteases.

## Author Contributions

**Daniel Guimarães Silva Paulo:** conceptualization, data curation, data collection and investigation, formal analysis, funding acquisition, project administration, resources, software, visualization, writing – original draft, writing – review and editing. **Julia Renata Schneider**, **Rafael Junior Andrade, Humberto Josué de Oliveira Ramos:** data collection and investigation, data curation, formal analysis, writing – original draft. **Ian Lucas Batista Santos, Cibele Cunha Vilela, Maria Clara Neves Gomes Rodrigues, Milena Godoi Lima, Rafael de Almeida Barros, Neilier Rodrigues da Silva Júnior, Wesley Borges Wurlitzer**, **Yaremis Beatriz Meriño Cabrera, Noeli Juarez Ferla:** data collection and investigation. **Maria Goreti de Almeida Oliveira:** conceptualization, data curation, formal analysis, funding acquisition, project administration, resources, software, supervision, validation, visualization, writing – original draft, writing – review and editing.

## Conflicts of Interest

The authors declare no conflicts of interest.

## Supporting information


**Figure S1:** Pharmacophoric profile using Dyscovey studio programm between lepidopteran trypsin‐like proteases and the peptides AVIMK, AVIMR, TGPCK and TGPCR, obtained by molecular docking analysis. **Figure S2:** Types of chemical bonds formed between the peptides and multiple trypsin‐like isoforms of lepidopteran insects. Pie charts represent the relative contribution (%) of each interaction type predicted by molecular docking.
