# Supplementary material for: Peptides Derived From Reactive Center Loops Inhibit Digestive Trypsin‐Like Enzymes in Lepidopteran Pests
Source: Arch Insect Biochem Physiol. 2026 Jan 9;121(1):e70123. doi: 10.1002/arch.70123 (PMC12784448; doi:10.1002/arch.70123)

**SUPPLEMENTARY FILES**

**Figure S1**. Pharmacophoric profile using Dyscovey studio programm between lepidopteran trypsin-like proteases and the peptides AVIMK, AVIMR, TGPCK and TGPCR, obtained by molecular docking analysis.


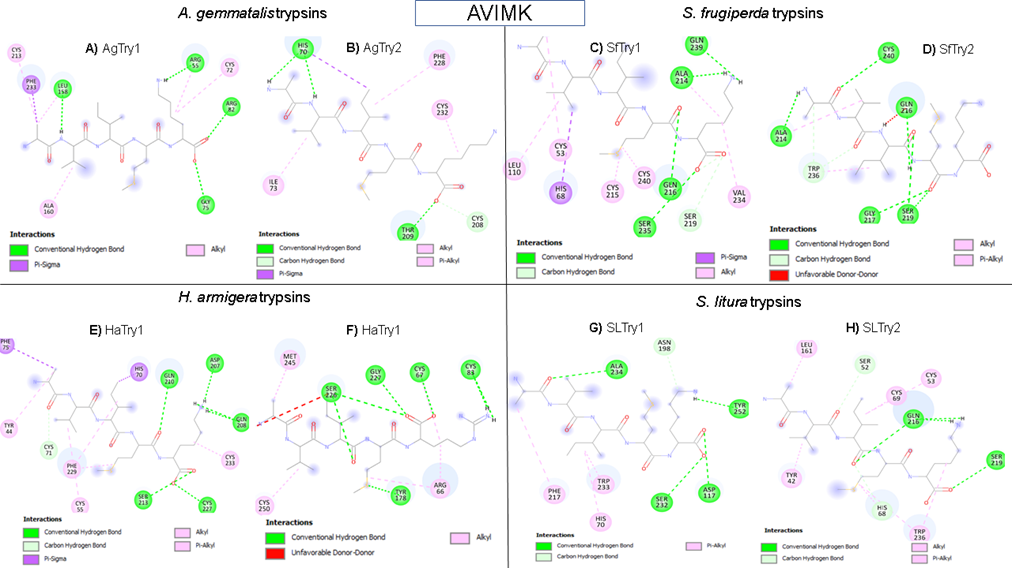


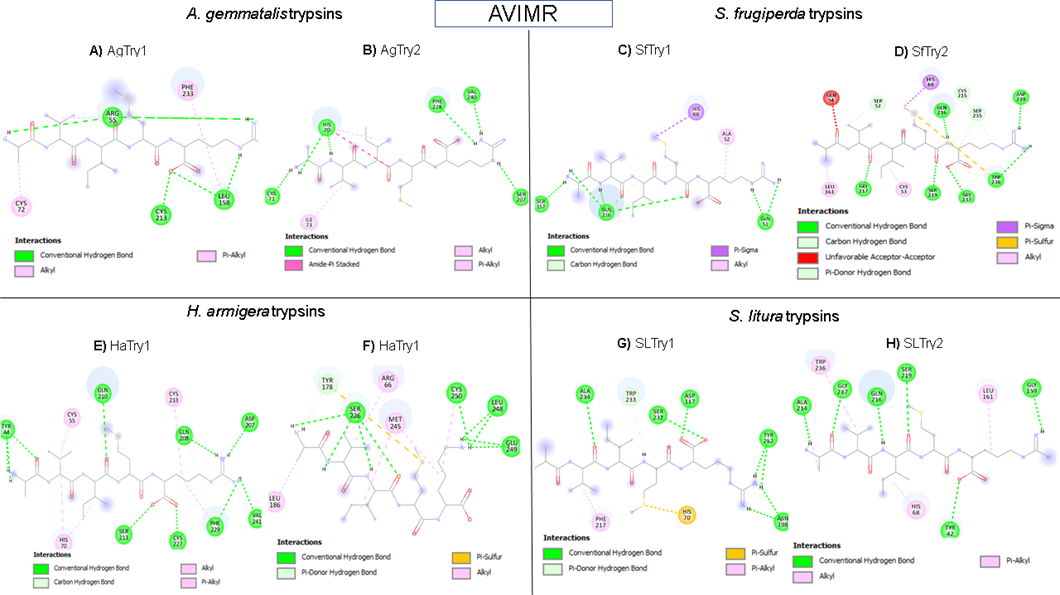


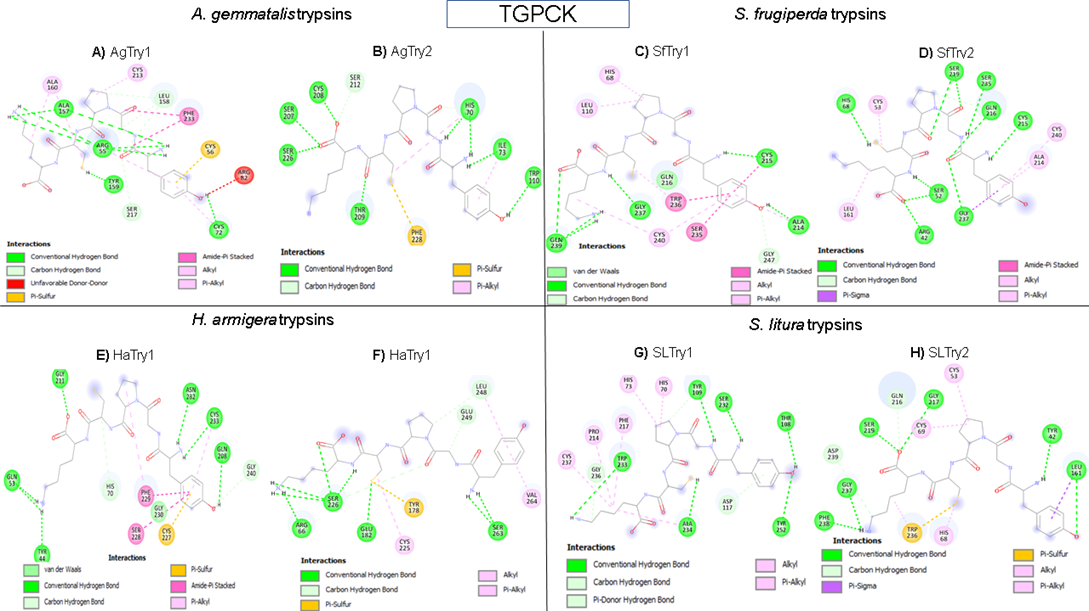


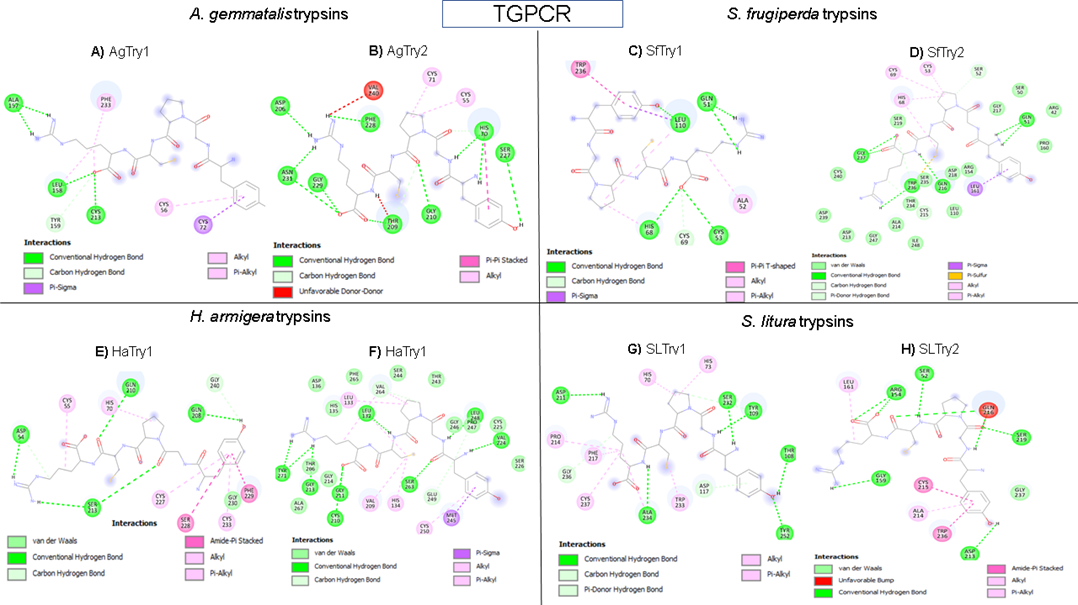


**Figure S2**. Types of chemical bonds formed between the peptides and multiple trypsin-like isoforms of lepidopteran insects. Pie charts represent the relative contribution (%) of each interaction type predicted by molecular docking.


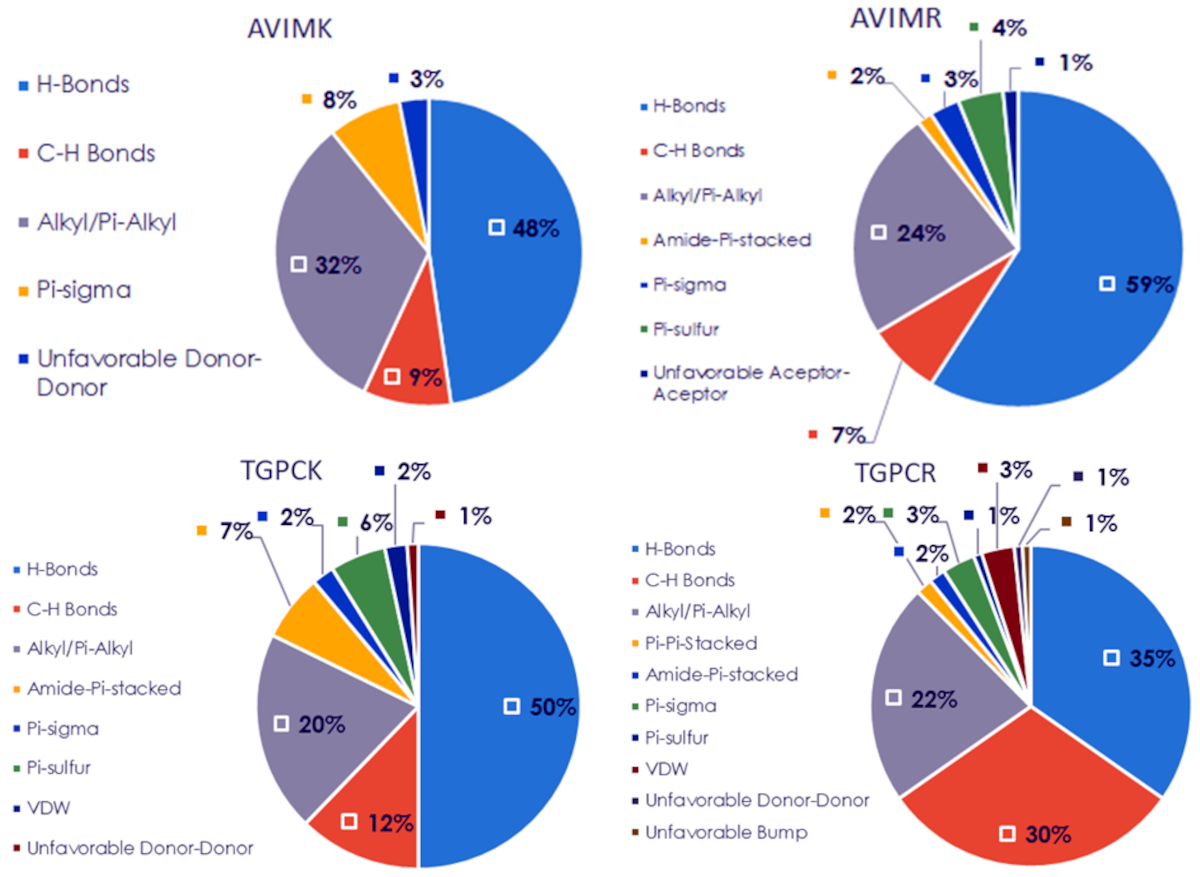

Supplement: Supplementary file 1 — Figure S1: Pharmacophoric profile using Dyscovey studio programm between lepidopteran trypsin‐like proteases and the peptides AVIMK, AVIMR, TGPCK and TGPCR, obtained by molecular docking analysis. Figure S2: Types of chemical bonds formed between the peptides and multiple trypsin‐like isoforms of lepidopteran insects. Pie charts represent the relative contribution (%) of each interaction type predicted by molecular docking. [file ARCH-121-e70123-s001.docx]
